# Porcine lung mesenchymal stromal cells possess differentiation and immunoregulatory properties

**DOI:** 10.1186/s13287-015-0220-0

**Published:** 2015-11-11

**Authors:** Mahesh Khatri, Timothy D. O’Brien, Kuldeep S. Chattha, Linda J. Saif

**Affiliations:** Department of Food Animal Health Research Program, Ohio Agricultural Research and Development Center, The Ohio State University, 1680 Madison Avenue, Wooster, OH 44691 USA; Department of Veterinary Population Medicine, College of Veterinary Medicine, University of Minnesota, St. Paul, MN USA

**Keywords:** Lung mesenchymal stem cells, Acute lung injury, Stem cell, Large animal model

## Abstract

**Introduction:**

Mesenchymal stem (stromal) cells (MSCs) possess self-renewal, differentiation and immunoregulatory properties, and therefore are being evaluated as cellular therapy for inflammatory and autoimmune diseases, and for tissue repair. MSCs isolated from bone marrow are extensively studied. Besides bone marrow, MSCs have been identified in almost all organs of the body including the lungs. Lung-derived MSCs may be more effective as therapy for lung diseases as compared to bone marrow-derived MSCs. Pigs are similar to humans in anatomy, physiology and immunological responses, and thus may serve as a useful large animal preclinical model to study potential cellular therapy for human diseases.

**Methods:**

We isolated MSCs from the lungs (L-MSCs) of 4–6-week-old germ-free pigs. We determined the self-renewal, proliferation and differentiation potential of L-MSCs. We also examined the mechanisms of immunoregulation by porcine L-MSCs.

**Results:**

MSCs isolated from porcine lungs showed spindle-shaped morphology and proliferated actively in culture. Porcine L-MSCs expressed mesenchymal markers CD29, CD44, CD90 and CD105 and lacked the expression of hematopoietic markers CD34 and CD45. These cells were multipotent and differentiated into adipocytes, osteocytes and epithelial cells. Like human MSCs, L-MSCs possessed immunoregulatory properties and inhibited proliferation of T cells and interferon-γ and tumor necrosis factor-α production by T cells and dendritic cells, respectively, and increased the production of T-helper 2 cytokines interleukin (IL)-4 and IL-13 by T cells. L-MSCs induced the production of prostaglandin E2 (PGE2) in MSC–T cell co-cultures and inhibition of PGE2 significantly restored (not completely) the immune modulatory effects of L-MSCs.

**Conclusions:**

Here, we demonstrate that MSCs can be isolated from porcine lung and that these cells, similar to human lung MSCs, possess in vitro proliferation, differentiation and immunomodulatory functions. Thus, these cells may serve as a model system to evaluate the contribution of lung MSCs in modulating the immune response, interactions with resident epithelial cells and tissue repair in a pig model of human lung diseases.

## Introduction

Mesenchymal stem (stromal) cells (MSCs) are multipotent cells that were first identified in bone marrow (BM) as plastic adherent fibroblast cells. Subsequent studies suggested that MSCs exist in almost all tissues including the lungs [1, 2]. MSCs express mesenchymal markers such as CD44, CD73, CD90, and CD105, and are negative for the expression of hematopoietic markers CD34 and CD45 [3]. These cells are multipotent and are capable of differentiating into multiple cell types including adipocytes, osteocytes, chondrocytes, neurons, cardiomyocytes and epithelial cells [4–7].

MSCs possess immunomodulatory properties and inhibit inflammation and immunological responses. Furthermore, MSCs express low levels of major histocompatibility complex molecules and do not express co-stimulatory molecules such as CD40, CD80 and CD86 that mediate T activation; hence these cells escape lysis by T cells. Many reports indicated that MSC-mediated immunosuppressive effects are due to the release of soluble factors [8]. Due to their proliferative capacity, differentiation and immunoregulatory properties, MSCs are attractive as cellular therapy for inflammatory and autoimmune diseases, and regenerative medicine.

Mice are extensively used as an animal model for human diseases due to their easy handling. Undoubtedly, mouse models have contributed immensely to our understanding of the pathogenesis of human diseases and in providing useful insights into the mechanisms of stem cell-mediated beneficial effects for human diseases [9]. However, results obtained from the rodent models are not always reproducible in humans [10, 11]. In addition, due to differences in the size and physiology of mouse and human organs, it is difficult to evaluate the functional consequences of cell therapy in mouse models. Moreover, the short life span of mice does not allow evaluation of long-term effects of stem cell therapy. These differences highlight the need for confirming the results obtained from mouse models in large animal models, such as pigs, before testing stem cell therapy in human clinical trials. Pigs offer several advantages as a large animal model for stem cell therapy for human conditions/diseases. Similar to humans, pigs are an out-bred species and they resemble humans in anatomy, physiology, and immune responses [12–17]. Moreover, pigs are similar to humans in terms of body size and have a fairly long life span which makes them an ideal large animal model for cellular therapy [18–22].

In this study, we isolated and characterized MSCs from the lungs of pigs (L-MSCs). Additionally, we examined the mechanisms of immunoregulation by L-MSCs and observed prostaglandin E2 (PGE2) as one of the mediator likely involved in the immunomodulatory activity of L-MSCs. L-MSCs described here are similar to human L-MSCs in terms of phenotype, differentiation and immunoregulation; therefore, these cells will be useful in elucidating the mechanisms of MSC-mediated therapeutic effects in a pig model of human lung diseases.

## Methods

### Isolation and culture of porcine L-MSCs

Near-term pregnant Landrace-Yorkshire white-Duroc crossbred sows (Swine herd, The Ohio State University, Columbus, USA) were delivered by hysterectomy and piglets were housed in sterile gnotobiotic isolator units as described previously [23]. The piglets of either sex were reared and euthanized according to the protocols approved by the Institutional Laboratory Animal Care and Use Committee, The Ohio State University. BM and lungs were harvested from 4–6-week-old germ-free pigs (n = 6). MSCs from BM were isolated as described earlier [24, 25] and these cells were included as positive controls for analyzing the expression of mesenchymal markers on MSCs. Lung tissues were cut into small pieces (approximately 3–4 cm) and treated with 0.5 mg/ml collagenase type II (Life Technologies) in Dulbecco’s modified Eagle’s medium (DMEM) for 1 h at 37 °C with continuous shaking. The single cell suspensions were filtered through 70-μm filters (BD, Falcon, USA) and mononuclear cells were isolated by centrifugation on Ficoll-Hypaque (GE HealthCare). After washing, cells were resuspended in DMEM containing 10 % fetal bovine serum (FBS) and 1 % antibiotics. The cells were seeded in T-25 cm^2^ flasks at 1 × 10^5^/cm^2^. After 2–3 days of incubation, nonadherent cells were discarded and fresh medium was added to the cultures. The cells were trypsinized at confluency using 0.05 % trypsin-EDTA (Gibco) and were passaged at least 2 times to remove macrophages. MSCs passaged between 2 and 8 times were used in all the experiments.

Self-renewal potential of L-MSCs was assessed by colony forming unit-fibroblast (CFU-F) assay as described previously [24]. Briefly, 100 L-MSCs/well were seeded in a six-well plate. The cells were incubated at 37 °C for 10 days with fresh media added every 3 days. After 10 days of incubation, cells were stained with Giemsa stain and colonies greater than 3 mm were counted.

### Cell proliferation assay

Proliferation potential of L-MSCs was determined by MTT assay as described previously [26]. Ten thousand cells suspended in 100 μl DMEM were plated in each well of flat-bottomed 96-well plates. At indicated times, 20 μl MTT (2 mg/ml) was added to each well and cells were incubated further for 4 h at 37 °C. After the incubation, medium was removed and 100 μl DMSO was added and plates were incubated for 30 min at 37 °C to dissolve blue formazan crystals. The absorbance was read at 570 nm with a reference filter of 620 nm on a plate reader (Spectramax; Molecular Device). Cell growth was measured by plotting optical density (OD) values against time.

### L-MSC phenotyping by flow cytometry

L-MSCs were examined for the expression of mesenchymal markers (CD29, CD44, CD90 and CD105), hematopoietic markers (CD34 and CD45), monocyte (CD14) B cell (CD79β) and swine leucocyte antigen (SLA) I and II by flow cytometry. BM-MSCs were included as positive controls for comparison. MSCs were stained with the following primary antibodies: mouse anti-pig CD29 (IgG1), mouse anti-pig CD90 (IgG1), mouse anti-pig CD44 (IgG2b, VMRD), mouse anti-pig CD45 (IgM, VMRD), mouse anti-pig/human CD105 (IgG2a, GeneTex), Mouse anti pig CD14 (IgG2b, Serotec), rat anti-pig CD79b (IgG1, Serotec), FITC Mouse Anti-Human CD34 (IgG1, BD Biosciences), SLA-I (IgG2a, VMRD), SLA-II (IgG2b, VMRD; IgG2b FITC, Serotec) for 20 min at 4 ° C. After washing, cells were stained with appropriate secondary antibodies. Cells were acquired using a C6 flow cytometer (BD Accuri Cytometers) and analyzed using CFlow® plus Software (Accuri) [27].

### Detection of octamer-binding transcription factor 4 expression on L-MSCs

L-MSCs were examined for the expression of pluripotency (octamer-binding transcription factor 4; Oct4) marker by immunofluorescence assay (IFA) [28, 29]. Porcine MSCs have been shown to express Oct4; therefore, BM-MSCs were included as positive control [30, 31]. The cells were fixed in a mixture of methanol:acetone (1:1) for 3 min at room temperature followed by blocking with 3 % bovine serum albumin for 30 min. Cells were incubated at 4 °C overnight with rabbit anti-human Oct4 (Santa Cruz Biotechnology). Next, cells were incubated with FITC-labeled secondary antibody for 45 min at room temperature. Cell nuclei were stained with 4′,6-diamidino-2-phenylindole (DAPI).

### In vitro differentiation of L-MSCs into adipocytes and osteocytes

Differentiation of L-MSCs into adipocytes and osteocytes was examined as described previously by Khatri et al. [24]. In brief, for adipogenesis, L-MSCs at 70–80 % confluence were cultured in DMEM supplemented with 10 % FBS, 1 μM dexamethasone, 10 μg/ml insulin, and 0.2 mM indomethacin for 21 days. The medium was refreshed every 3–4 days. Adipogenic differentiation of L-MSCs was assessed by Oil Red O staining.

For osteogenic differentiation, cells were cultured for 21 days in DMEM containing 10 % FBS, 100 nM dexamethasone, 10 mM β-glycerophosphate (Sigma-Aldrich), and 0.05 mM L-ascorbic acid-2-phosphate (Sigma-Aldrich). Cells were fed with fresh medium every 3–4 days. Calcium deposition indicative of osteogenesis was detected by Von Kossa staining [24].

### In vitro differentiation of MSCs into epithelial phenotypes

L-MSCs were cultured in epithelial growth medium containing bovine pituitary extract (70 μg/ml), human epidermal growth factor (5 ng/ml), insulin (5 μg/ml), and hydrocortisone (0.5 μg/ml) (MEGM, Lonza, Walkersville, MD, USA) for 10 days. The expression of epithelial markers, pancytokeratin and cytokeratin-18 was detected by IFA using mouse anti-human pancytokeratin and anti-human cytokeratin-18 (Sigma) primary and appropriate secondary antibodies by IFA [28, 29].

### Immunomodulation by L-MSCs

L-MSCs were isolated from 4–6-week-old germ-free pigs (n = 6) as described above. Peripheral blood mononuclear cells (PBMCs) and dendritic cells (DCs) were isolated from conventional 4–6-week-old specific pathogen-free Large White-Duroc crossbred pigs (n = 3) and were used in L-MSC–PBMC–DC co-culture experiments.

#### MSC–DC co-cultures

BM-DCs were isolated from 4–6-week-old pigs as described previously with minor modifications [32]. Briefly, BM mononuclear cells were cultured in RPMI-1640 medium supplemented with 10 % FBS, 1 % antibiotics and 10 ng/ml each of recombinant swine granulocyte macrophage colony-stimulating factor and recombinant swine interleukin (IL)-4 for 3 days. DCs were identified as nonadherent cells showing dendrites and by the expression of SwC3a and SLA-II. DCs cultured in the presence or absence of MSCs were stimulated with lipopolysaccharide (LPS; 100 ng/ml) overnight. The levels of tumor necrosis factor (TNF)-α in culture supernatants were measured by enzyme-linked immunosorbent assay (ELISA) [27].

#### T-cell proliferation and Th1 and Th2 cytokine detection

T-cell proliferation was analyzed by staining the cells with carboxyfluorescein diacetate succinimidyl ester (CFSE). PBMCs were isolated from 4–6-week-old SPF pigs by Ficoll-Hypaque gradient separation [27]. PBMCs were labeled with 5 μM CFSE for 5 min at room temperature. After washing, the cells were suspended in RPMI 1640 medium supplemented with 10 % FBS and 1 % antibiotics (Gibco) in a 24-well plate in the presence or absence of MSCs (MSC to PBMC ratio 1:10). The cultures were stimulated with mitogen (phytohemagglutinin (PHA); 5 μg/ml) and incubated for 72 h at 37 °C. Cell proliferation was analyzed by flow cytometry (Accuri).

For the measurement of cytokines in MSC–PBMC co-cultures, PBMCs were stimulated with PHA (5 μg/ml) in the presence or absence of L-MSCs. At 24 h, supernatants were collected and interferon (IFN)-γ (T helper 1; Th1), IL-4 and IL-13 (Th2) levels were determined by ELISA as described previously [27].

The levels for IL-6 (proinflammatory), IL-10 (anti-inflammatory) and PGE2 in the cell-culture supernatant of MSC–PBMC co-cultures were also determined by ELISA and enzyme immunoassay (Cayman chemicals, AnnArbor, MI), respectively.

#### Induction of T regulatory cells in MSC–T cell co-cultures

PBMCs (1 × 10^6^) were cultured in the presence or absence of L-MSCs (1 × 10^5^) for 48 h at 37 °C. Induction of T regulatory cells (Tregs) in L-MSC–PBMC co-cultures was analyzed by examining the expression of regulatory T-cell marker (FoxP3) on CD4 cells by flow cytometry as described previously [33]. Briefly, cells isolated from co-cultures were stained with CD4-PE. Cells were then fixed and permeabilized with Cytofix/Cytoperm buffer (BD Biosciences) according to manufacturer’s instructions followed by staining with rat anti-mouse Foxp3-FITC Ab (clone FJK-16 s; eBioscience). Appropriate isotype controls were also included during staining. Cells were acquired using a C6 flow cytometer (BD Accuri Cytometers) and analyzed using CFlow® plus Software (Accuri) [27].

### Effect of PGE2 inhibition on T-cell proliferation

To examine the effect of PGE2 inhibitor on T-cell proliferation, L-MSCs were incubated overnight in the presence or absence of PGE2 inhibitor NS-398 (Cayman chemicals) [34]. PBMCs isolated from out-bred conventional pigs were stained with CFSE. PBMCs were then added to MSC cultures (1:10 ratio) and both cell types were cultured in media containing NS-398 (5 μM). PBMCs cultured in the presence or absence of MSCs were stimulated with PHA for 72 h. Cell proliferation was analyzed by flow cytometry. We also examined the effect of PGE2 inhibition on TNF-α and IFN-γ production by DCs and PBMCs, respectively. L-MSCs were cultured overnight in medium with or without NS-398. DCs or PBMCs suspended in RPMI medium with or without NS-398 were added to the L-MSCs. MSC/DC cultures were stimulated with LPS (100 ng/mL) and MSCs/PBMC cultures were stimulated with PHA (5 μg/mL). After 24-h incubation, TNF-α production in MSC/DC and IFN-γ in MSC/T cell co-cultures was determined by ELISA.

### Statistical analysis

Data were expressed as the mean ± SD from three independent experiments/three or more pigs. A two-tailed Student *t-*test or one-way analysis of variance followed by Tukey test was used to compare significant differences between groups. A *P* value <0.05 was considered to be statistically significant.

## Results

### Isolation of plastic-adherent porcine L-MSCs

**Fig. 1:**
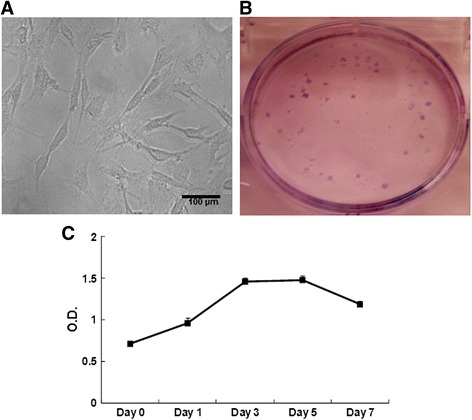
Characteristics of porcine L-MSCs. **a** Morphology of porcine L-MSCs. Porcine L-MSCs exhibit characteristic fibroblast-like morphology. **b** Colony forming unit-fibroblast assay. L-MSCs were cultured at 100 cells/well in a six-well plate. Single cells proliferated and formed colonies as shown by Giemsa staining. **c** In vitro proliferation potential of L-MSCs. L-MSCs were suspended in DMEM containing 10 % FBS and cultured in a 96-well plate. At indicated intervals, cell proliferation was measured by MTT assay. Optical density (*OD*) values at each time point are mean ± SD of L-MSCs isolated from three different pigs and cultured in triplicate wells

MSCs were successfully isolated from the lungs of all six pigs. These MSCs showed characteristic features of MSCs, such as adherence to plastic surface and fibroblast-like morphology (Fig. 1a).

One important characteristic of MSCs is their ability to self-renew and proliferate. The self-renewal potential of MSCs was assessed by CFU-F assay. One hundred L-MSCs cultured in each well of a six-well plate formed 28 ± 8 (n = 6) colonies indicating their self-renewal ability (Fig. 1b). L-MSCs possessed high in vitro proliferation capacity as shown in Fig. 1c.

### Porcine L-MSCs express mesenchymal and pluripotency markers

**Fig. 2:**
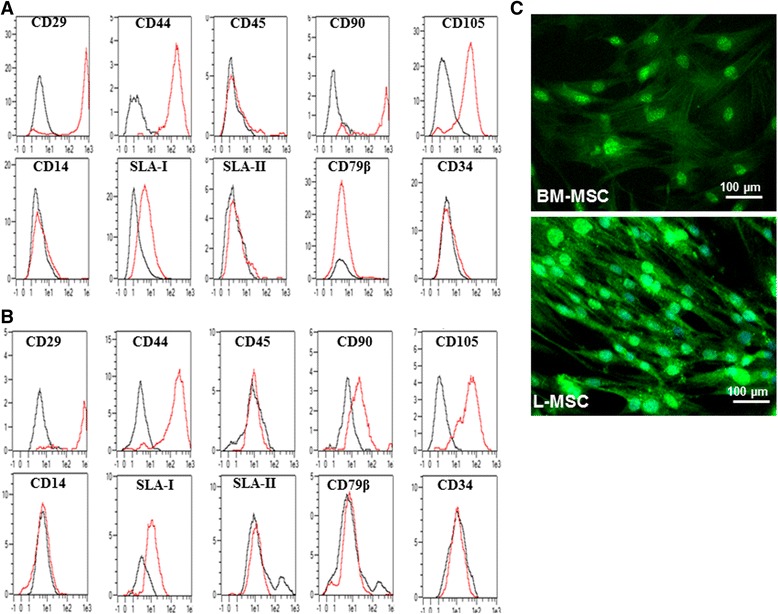
Phenotype analysis of porcine L-MSCs and expression of pluripotency markers. Porcine (**a**) BM**-**MSCs and (**b**) L-MSCs were detached by trypsinization and single cell suspension was examined for the expression of mesenchymal markers (CD29, CD44 CD90 and CD105), hematopoietic (CD34 and CD45), CD14, CD79β, SLA-I and SLA-II by flow cytometry. *Black line* isotype control, *red line* antibody staining. (**c**) Expression of Oct4 on L-MSCs. L-MSCs were examined for the expression of the pluripotency marker, Oct4, by IFA. BM-MSCs were included as positive control. *BM-MSC* Bone marrow mesenchymal stem cell, *L-MSC* Lung mesenchymal stem cell, *SLA* Swine leucocyte antigen

Colony-expanded L-MSCs and BM-MSCs were examined for the expression of mesenchymal markers by flow cytometry. BM- and L-MSCs showed the expression of CD29, CD44, CD 90 and CD105 but were negative for CD34, CD45, CD14 and CD79β suggesting the mesenchymal lineage of these cells (Fig. 2a and b ). These cells also expressed moderate levels of SLA-1 but not SLA-II (Fig. 2a and b ).

L-MSCs were also examined for the expression of the pluripotency marker Oct4 (Fig. 2c). The expression of Oct4 was mainly detected in the cell nuclei of L-MSCs.

### Porcine L-MSCs can differentiate into adipocytes, osteocytes and epithelial cells

MSCs from BM and other anatomical locations demonstrate mutilineage differentiation potential. L-MSCs also demonstrated mutilineage differentiation potential.

**Fig. 3:**
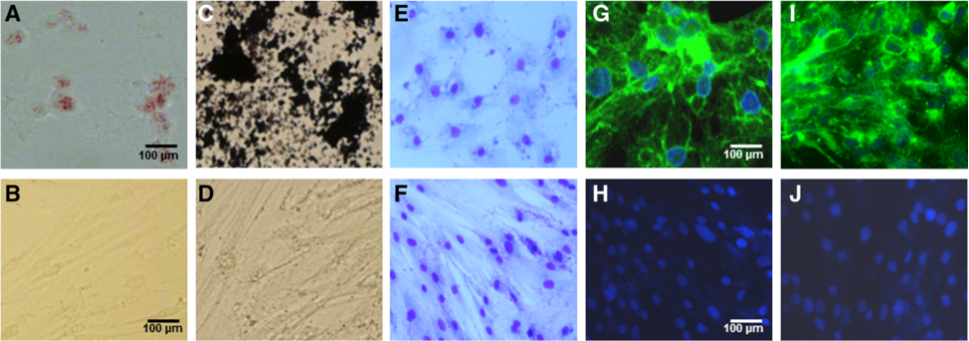
Differentiation potential of porcine L-MSCs. **a** Adipocyte differentiation. L-MSCs when cultured in adipogenic medium for 21 days showed lipid droplets in the cytoplasm of differentiated cells. **b** No adipocyte differentiation was detected in cells cultured in DMEM. **c** Osteocyte differentiation. L-MSCs cultured in osteogenic medium for 21 days showed calcium deposition as detected by Von Kossa staining. **d** No osteogenic differentiation was observed in cells cultured in DMEM. **e**–**h** Epithelial differentiation. L-MSCs cultured in epithelial differentiation medium for 10 days exhibited cuboidal morphology (**e**) and were found to express epithelial markers pan-cytokeratin (**g**) and cytokeratin-18 (**i**) whereas L-MSCs cultured in DMEM displayed normal spindle-shaped morphology (**f**), and expression of pan-cytokeratin (**h**) and cytokeratin-18 (**j**) was not detected on undifferentiated L-MSCs

L-MSCs when cultured in adipocyte induction media for 21 days differentiated into adipocytes. Differentiated cells contained multiple lipid vacuoles as demonstrated by staining with Oil Red O (Fig. 3a). Incubation of L-MSCs in osteogenic media for 3 weeks demonstrated tightly packed nodule-like structures. Calcium deposition in differentiated cells was detected by Von Kossa staining (Fig. 3c).

L-MSCs also differentiated into epithelial cells. L-MSCs cultured in epithelial cell differentiation media for 10 days exhibited cuboidal-like morphology (Fig. 3e) and positive staining for epithelial cell markers pancytokeratin and cytokeratin-18 (Fig. 3g and i).

### Immunomodulation by L-MSCs

#### L-MSCs inhibit TNF-α secretion by DCs

**Fig. 4:**
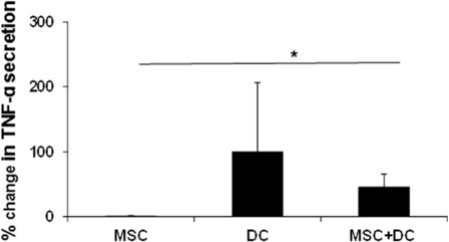
L-MSCs suppress TNF-α secretion by DCs. Porcine BM-DCs were cultured in the presence or absence of L-MSCs and stimulated with LPS overnight. TNF-α production in culture supernatants was determined by ELISA. Presence of L-MSCs in the culture caused more than 50 % suppression in TNF-α production by LPS-stimulated DCs. The data are expressed as % change in TNF-α production (TNF-α produced by LPS-stimulated DCs was considered as 100 %). Each bar represents mean percent change ± SD in TNF-α levels from three independent experiments. *P < 0.05. *DC* Dendritic cell, *MSC* Mesenchymal stem cell, *TNF* tumor necrosis factor

L-MSCs were co-cultured with BM-derived DCs at a ratio of 1:10 and stimulated with LPS overnight. Data are expressed as percent change in TNF-α production in DCs in the presence or absence of MSCs (Fig. 4). There was more than a 50 % decrease in TNF-α production in DC-MSC co-cultures as compared to DCs alone. However, these differences did not reach a significant level due to large variations in the levels of TNF-α production between individual pigs. L-MSCs alone did not produce detectable levels of TNF-α.

#### L-MSCs suppress T-cell proliferation

**Fig. 5:**
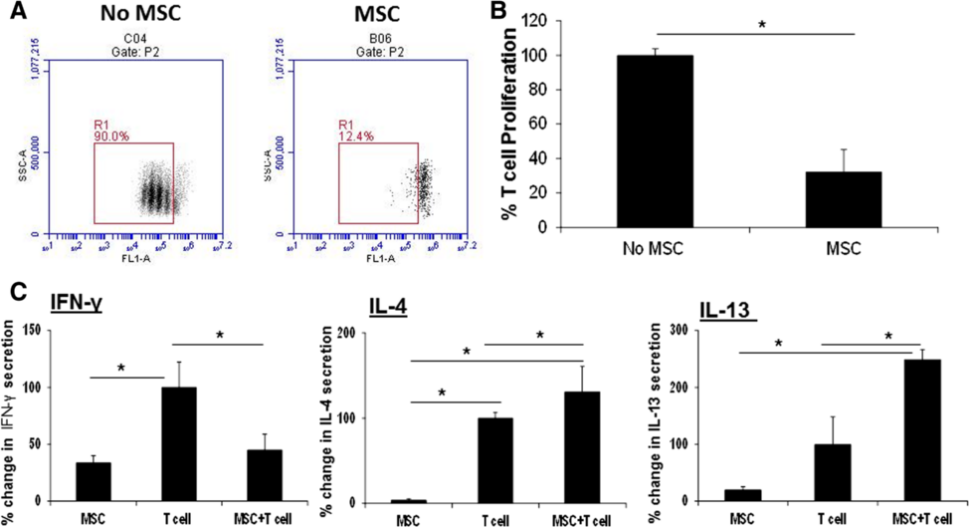
L-MSCs suppress mitogen-stimulated T-cell proliferation and Th1 cytokine secretion and promote Th2 cytokine secretion by T cells. **a** L-MSCs inhibit mitogen-stimulated T-cell proliferation. L-MSCs (1 × 10^5^/well) were plated in a 24-well plate overnight. CFSE-labeled PBMCs (1 × 10^6^/well) isolated from unrelated pigs were added to L-MSC cultures and stimulated with PHA (5 μg/ml). L-MSC–PBMC co-cultures were incubated for 72 h at 37 °C. The cell proliferation was analyzed by flow cytometry. A representative flow diagram is shown. **b** Data are presented as % change in proliferating T cells relative to PHA-stimulated PBMCs without MSC group (PBMCs stimulated with PHA were considered as 100 %). The experiment was performed with L-MSCs/PBMCs isolated from three different pig pairs. ^*^ *P* < 0.05, versus the no MSC group. **c** L-MSCs inhibit Th1 and enhance Th2 cytokine production by T cells. PBMCs were stimulated with PHA in the presence or absence of L-MSCs for 24 h. After the incubation, the supernatants were collected and examined for Th1 (IFN-γ) and Th2 (IL-4 and IL-13) cytokine production by ELISA. Data are expressed as % change in IFN-γ, IL-4 and IL-13 production in L-MSC/PBMC co-cultures relative to PHA-stimulated PBMCs (PBMCs stimulated with PHA considered as 100 %). The values are means ± SD. *P < 0.05. The experiments were performed with L-MSCs and PBMCs isolated from three germ-free and SPF pigs respectively. *IFN* Interferon, *IL* Interleukin, *MSC* Mesenchymal stem cell

Previous reports have shown that MSCs suppress T-cell proliferation [34–36]. Therefore, we examined the effect of the L-MSCs on proliferation of T cells. In vitro expanded L-MSCs from three different germ-free pigs were co-cultured with PBMCs isolated from three pigs. L-MSCs were highly immunosuppressive and significantly (*P* < 0.05) inhibited PHA-induced T-cell proliferation (Fig. 5a).

Next we examined whether MSCs modulate the cytokine secretion by mitogen-stimulated PBMCs. PBMCs stimulated with PHA produced significant amounts of IFN-γ. However, in the presence of L-MSCs, there was a significant (*P* < 0.05) decrease in IFN-γ production and an increase in IL-4 and IL-13 production by stimulated PBMCs (Fig. 5b). These data suggest that MSCs directed the T-cell response towards the Th2 phenotype.

#### L-MSC–PBMC co-cultures produce cytokines

**Fig. 6:**
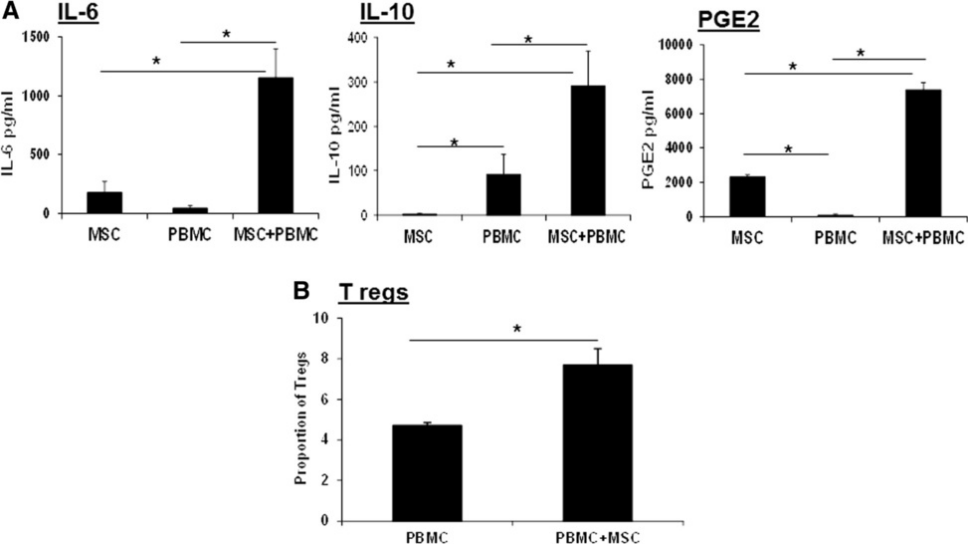
L-MSCs secrete soluble factors. **a** L-MSCs were co-cultured with PBMCs (1:10 ratio) for 24 h. After the incubation, levels of IL-6, IL-10 and PGE2 in culture supernatants were measured by ELISA. Data are presented as mean ± SD of IL-6, IL-10 and PGE2 levels in pg/ml in MSC–PBMC co-cultures, each cell type isolated from three different pig pairs. *P < 0.05. **b** MSCs promote the induction of Tregs. PBMCs (1 × 10^6^) were cultured in the presence or absence of L-MSCs (1 × 10^5^) for 48 h. Expression of regulatory T-cell marker (FoxP3) on CD4 cells was analyzed by flow cytometry. In the presence of L-MSCs, an increased proportion of Tregs was induced. Data are presented as mean ± SD proportion of CD4^+^FoxP3^+^cells. L-MSCs and PBMCs were isolated from three different pigs. *IL* Interleukin, *MSC* Mesenchymal stem cell, *PBMC* Peripheral blood mononuclear cell, *PGE2* Prostaglansin E2, *Tregs* T regulatory cells

MSCs isolated from humans have been shown to modulate innate and adaptive immune cell functions by the secretion of soluble factors [37–39]. We detected the constitutive expression of IL-6 and PGE2 in L-MSC culture supernatants. L-MSCs also produced small quantities of IL-10. However, when PBMCs were added to L-MSCs cultures, a significant increase (*P <* 0.05) in levels of these factors was observed (Fig. 6a).

We detected significant levels of IL-10 in MSC-PBMC co-cultures. Therefore, we wanted to see whether L-MSCs induce the induction of Tregs. There was a significant (*P <* 0.05) increase in the proportion of Tregs (FoxP3 expressing CD4 cells) when PBMCs were co-cultured with L-MSCs (Fig. 6b).

### Inhibition of PGE2 restores partial but significant T-cell proliferation and IFN-γ and TNF-α production

**Fig. 7:**
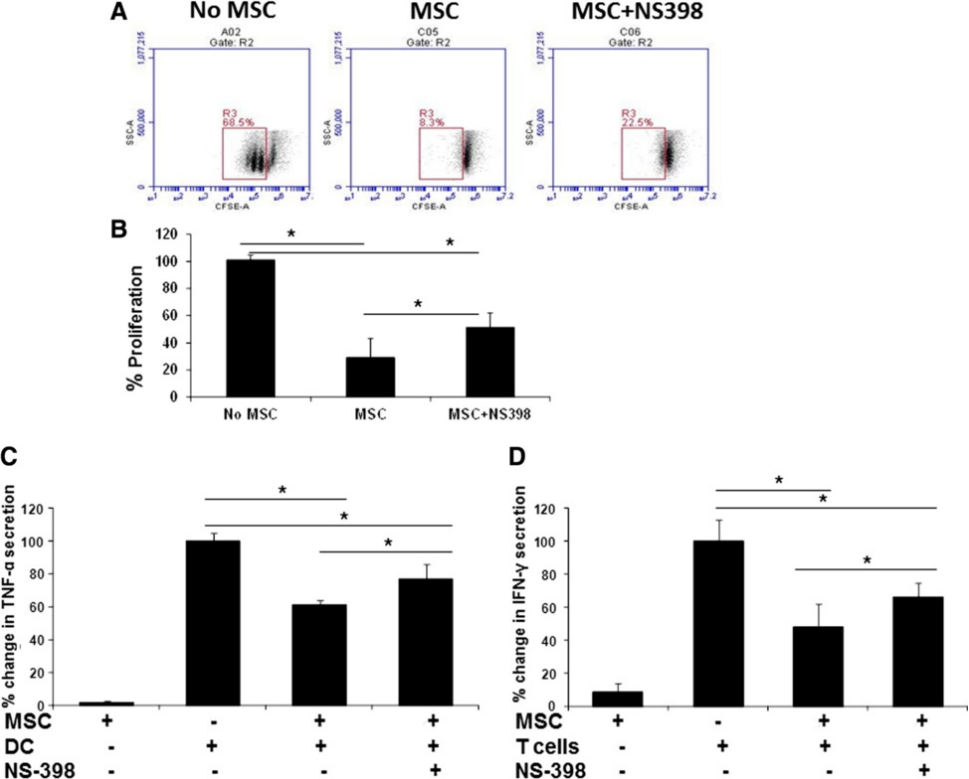
Immunomodulatory effects of L-MSCs are mediated via secretion of PGE2. **a** L-MSCs were cultured in media containing the PGE2 inhibitor NS-398 overnight. CFSE-labeled PBMCs from unrelated pigs were co-cultured with MSCs in media containing PGE2 inhibitor NS-398. The cultures were stimulated with PHA (5 μg/ml). After 72 h, cell proliferation was analyzed by flow cytometry. A representative flow diagram is shown. **b** Percent change in cell proliferation in the presence or absence of PGE2 inhibitor (cultures of PBMCs only stimulated with PHA were considered as 100 %). Data are presented as % change in cell proliferation (mean ± SD) from MSCs/PBMCs isolated from three unrelated pig pairs. *P < 0.05. **c** Overnight cultures of L-MSCs and DCs were co-cultured with or without NS-398 and stimulated with LPS. TNF-α secretion in culture supernatants was detected by ELISA. Addition of PGE2 inhibitor resulted in a significant increase (*P* < 0.05) in TNF-α levels. Data are presented as mean ± SD of TNF-α levels in MSCs/DCs isolated from three unrelated pig pairs. *P < 0.05. **d** L-MSCs and PBMCs were co-cultured in media with or without NS-398. The cultures were stimulated with PHA and incubated for 24 h. IFN-γ production in culture supernatants was measured by ELISA. There was an increase in IFN-γ levels in cultures incubated with NS-398 PGE2. Data are presented as mean ± SD of IFN-γ levels in MSC/PBMC co-cultures isolated from three unrelated pig pairs. *P < 0.05. *CFSE* Carboxyfluorescein diacetate succinimidyl ester, *DC* Dendritic cell, *IFN* Interferon, *MSC* Mesenchymal stem cell, *TNF* Tumor necrosis factor

In previous studies conducted with human BM- and L-MSCs, PGE2 was found to modulate immune cell functions [34, 35]. Similar to these studies, we also detected PGE2 in L-MSC–PBMC co-cultures. We used NS-398, a PGE2 synthesis inhibitor, to examine whether PGE2 regulates the immunomodulatory activity of L-MSCs. The blockade of PGE2 synthesis resulted in a significant increase (*P* < 0.05) in the proportion of mitogen-stimulated proliferating cells (Fig. 7a and b). Furthermore, inhibition of PGE2 secretion also resulted in a significant increase (*P* < 0.05) in TNF-α and IFN-γ production by DCs and T cells, respectively (Fig. 7c and d). These findings indicated that, similar to human MSCs, porcine L-MSC-produced PGE2 is likely involved in the regulation of MSC-mediated immunomodulatory effects.

## Discussion

In this study, we isolated mesenchymal cells from the lungs of pigs. The isolated cells expressed transcription factors Oct4 and surface mesenchymal markers but lacked the expression of hematopoietic stem cell markers. L-MSCs also differentiated into adipocytes, osteocytes and epithelial cells. Importantly, L-MSCs showed immunomodulatory properties and inhibited TNF-α production by DCs and inhibited IFN-γ production and T-cell proliferation. Mechanistically, we found that PGE2 was partly involved in mediating the immunomodulatory activity of L-MSCs on DCs and T cells.

Multipotent L-MSCs have been isolated from human lungs [2, 40]. Similar to human L-MSCs, porcine L-MSCs expressed mesenchymal lineage markers whereas hematopoietic markers were not expressed on either human or porcine L-MSCs [2]. Isolated L-MSCs, like their human counterparts, were multipotent and differentiated into adipocytes, osteocytes and epithelial cells [2, 40]. Additionally, we detected the expression of Oct4 in L-MSCs. Oct4 regulates self-renewal and pluripotency of MSCs [41]. The expression of Oct4 in L-MSCs may be involved in regulating the pluripotency and self-renewal of these cells.

Although we have not directly compared the epithelial differentiation potential of lung- and BM-derived MSCs, it is likely that L-MSCs may have greater capacity to differentiate into lung epithelial cells. Tissue origin is known to govern the differentiation of MSCs. Recently, it was demonstrated that, compared to BM-MSCs, L-MSCs readily differentiated into epithelial cells [40]. In the current study also, L-MSCs under in vitro conditions differentiated into epithelial cells. Many studies have previously shown that transplantation of BM- and umbilical cord-derived MSCs differentiated into lung epithelial cells in a lung injury model [42–44]. In future in vivo studies, we will examine the differentiation and tissue repair potential of L-MSCs in lung injury or disease models in pigs.

In this study, presence of L-MSCs in DC cultures caused suppression of TNF-α secretion. Similar to our observations, human BM-MSCs also inhibited the secretion of TNF-α in MSC–DC co-cultures. Although, we have not analyzed the effects of L-MSCs on expression of co-stimulatory molecules on DCs in this study, earlier studies have shown that MSCs suppress the maturation of DCs, thus decreasing their ability to cause expansion of T cells [45, 46].

Several studies conducted with human- and mouse-derived MSCs have shown that Tregs are induced in MSC–T cell co-cultures [34, 36]. In this study, we also observed an increase in the proportion of FoxP3 expressing CD4 T cells in L-MSC–PBMC co-cultures. However, we observed lower expression of T-cell activation marker, CD25, on T cells co-cultured with MSCs (data not shown). Similar to our observations, Le Blanc et al [47] and Batten et al [36] also showed the inhibition of CD25 expression on T cells co-cultured with MSCs. Two main subsets of Tregs have been defined: CD4^+^CD25^hi^FoxP3^+^ and CD4^+^CD25^lo^FoxP3^+^. There is evidence that regulatory T-cell function of Tregs is associated with the expression of FoxP3, not the CD25 expression [48].

MSCs modulate the functions of immune cells through the secretion of soluble factors [38]. In previous studies, Jarvinen et al. [35] have shown that lung-derived human MSCs modulate immune responses by production of PGE2. In this study, we examined whether PGE2 is involved in L-MSC-mediated suppression of porcine DCs and T-cell functions. Porcine L-MSCs produced PGE2, and the addition of T cells to MSC cultures resulted in a significant increase in PGE2 production. Blocking of PGE2 secretion by an inhibitor significantly restored T-cell proliferation and IFN-γ and TNF-α production. However, in our studies, the restoration of T-cell proliferation and IFN-γ production by T cells and TNF-α production by DCs after PGE2 blockage was not complete suggesting that other soluble factors may also be involved in MSC-mediated inhibition of T cells. Further studies are required to understand completely the mechanisms of T-cell inhibition by L-MSCs and to identify additional soluble factors that may have immunomodulatory activity on innate and adaptive immune cells.

## Conclusions

In conclusion, for the first time we isolated MSCs from the lungs of pigs. Porcine L-MSCs showed proliferation and multilineage differentiation and they possessed potent immunomodulatory activity. Identification and characterization of these cells will help in understanding the mechanisms of MSC-mediated attenuation of disease and lung repair in the clinically relevant pig model of human respiratory diseases.

## Abbreviations

BM: Bone marrow
CFSE: Carboxyfluorescein diacetate succinimidyl ester
CFU-F: Colony forming unit-fibroblast
DAPI: 4′,6-diamidino-2-phenylindole
DC: Dendritic cell
DMEM: Dulbecco’s modified Eagle’s medium
ELISA: Enzyme-linked immunosorbent assay
FBS: Fetal bovine serum
IFA: Immunofluorescence assay
IFN: Interferon
IL: Interleukin
L-MSC: Lung mesenchymal stem (stromal) cell
LPS: Lipopolysaccharide
MSC: Mesenchymal stem (stromal) cell
Oct4: Octamer-binding transcription factor 4
OD: Optical density
PBMC: Peripheral blood mononuclear cell
PGE2: Prostaglandin E2
PHA: Phytohemagglutinin
SLA: Swine leucocyte antigen
Th: T helper
TNF: Tumor necrosis factor
Tregs: T regulatory cells
